# Pediatric Nephrology: Highlights for the General Practitioner

**DOI:** 10.1155/2012/270725

**Published:** 2012-08-22

**Authors:** Mouin Seikaly, Sabeen Habib, Amin J. Barakat, Jyothsna Gattineni, Raymond Quigley, Dev Desi

**Affiliations:** ^1^Louisiana State University Health Sciences Center, Pediatric Nephrology, Shreveport, LA, USA; ^2^Louisiana State University Health Sciences Center, Shreveport, LA 71115, USA; ^3^Georgetown University Medical Center, Washington, DC 20007, USA; ^4^University of Texas Southwestern, Dallas, TX 75390, USA

The current special issue is written as a practical guide for the health care providers managing children with kidney diseases. In this issue, the guest editors have emphasized clinical skills, diagnostic procedures, treatment, long-term followup as well as underlying pathophysiology with the general practitioner in mind. Since the call for manuscripts by the journal, we received 27 excellent submissions. We selected 9 papers to include in this special issue. We chose only those that we deemed appropriate to the scope that we outlined for this special issue. 

The guest editors who are leading experts in the field of pediatric nephrology did a terrific job writing their designated sections. A. J. Barakat's excellent perspective on presentation of children with renal disease is a practical guideline for referral to the specialist. S. Habib reviews controversies in the management of urinary tract infection. R. Quigley provides a comprehensive yet concrete review about children with chronic kidney disease. J. Gattineni discusses thoroughly the management of children presenting with proteinuria and hematuria. In my section on hypertension, I tried to provide a comprehensive practical guide that can be used in the management of a child with elevated blood pressure.

The nine papers that we selected for publication were outstanding. Y. R. Bhat et al. discuss the antenatal Bartter syndrome in a succinct fashion. D. Morin et al. provide a detailed review on nephrogenic SIADH, and kidney disease and type 2 diabetes mellitus are very well discussed by A. B. Dart et al. The approach to a patient with acute glomerulonephritis is also well covered by T. R. Welch. 

On behalf of the contributing authors, we hope that the readership will use our contribution to help them manage children with kidney disease.

